# Environmental Assessment of Housing for Older Adults Facing Housing Insecurity

**DOI:** 10.1093/geroni/igab046.2039

**Published:** 2021-12-17

**Authors:** Shreemouna Gurung, Shelby Elkes, Atiya Mahmood, Muhammad Qureshi, Habib Chaudhury, Sarah Canham, Rachel Weldrick

**Affiliations:** 1 Simon Fraser University, Vancouver, British Columbia, Canada; 2 University of Utah, Salt Lake City, Utah, United States

## Abstract

The Aging in the Right Place Environmental Audit (AIRP-ENV) and Secondary Observation (AIRP-ENV-SO) tools were developed to conduct observation-based audit of the built environment in shelters, transitional housing, independent housing with offsite/onsite supports, and permanent supportive housing with onsite medical and/or specialized services for older adults experiencing (or at risk of) homelessness. The 241 item AIRP-ENV tool is used to audit the presence/absence of exterior and interior built environmental features that support housing stability. The seven open-ended questions in the AIRP-ENV-SO tool is used to collect contextual data on function, safety and land-use of surrounding neighborhood. Data were collected at four sites of a transitional housing program in Vancouver, Canada as part of a multi-year, multi-city partnership project on aging and homelessness. Preliminary results demonstrate that built environment and urban design features (e.g., access, privacy, flexible and supportive spaces) contribute towards tenants’ residential resiliency and aging in place.

